# Effectiveness of Medicinal Plants for Glycaemic Control in Type 2 Diabetes: An Overview of Meta-Analyses of Clinical Trials

**DOI:** 10.3389/fphar.2021.777561

**Published:** 2021-11-26

**Authors:** Merlin L. Willcox, Christina Elugbaju, Marwah Al-Anbaki, Mark Lown, Bertrand Graz

**Affiliations:** ^1^ Primary Care Research Centre, Aldermoor Health Centre, University of Southampton, Southampton, United Kingdom; ^2^ Medicines Unit, Antenna Foundation, Geneva, Switzerland

**Keywords:** type 2 diabetes mellitus, phytomedicines, medicinal plants, herbal preparations, metaanalysis, randomised controlled clinical trials, glycaemic control, HbA1c

## Abstract

**Aims:** To rank the effectiveness of medicinal plants for glycaemic control in Type 2 Diabetes (T2DM).

**Methods:** MEDLINE, EMBASE, CINAHL and Cochrane Central were searched in October 2020. We included meta-analyses of randomised controlled clinical trials measuring the effectiveness of medicinal plants on HbA1c and/or Fasting Plasma Glucose (FPG) in patients with T2DM.

**Results:** Twenty five meta-analyses reported the effects of 18 plant-based remedies. Aloe vera leaf gel, Psyllium fibre and Fenugreek seeds had the largest effects on HbA1c: mean difference –0.99% [95% CI−1.75, −0.23], −0.97% [95% CI −1.94, −0.01] and −0.85% [95% CI −1.49, −0.22] respectively. Four other remedies reduced HbA1c by at least 0.5%: *Nigella* *sativa*, *Astragalus membranaceus,* and the traditional Chinese formulae Jinqi Jiangtang and Gegen Qinlian. No serious adverse effects were reported. Several other herbal medicines significantly reduced FPG. Tea and tea extracts (*Camellia sinensis*) were ineffective. However, in some trials duration of follow-up was insufficient to measure the full effect on HbA1c (<8 weeks). Many herbal remedies had not been evaluated in a meta-analysis.

**Conclusion:** Several medicinal plants appear to be as effective as conventional antidiabetic treatments for reducing HbA1c. Rigorous trials with at least 3 months’ follow-up are needed to ascertain the effects of promising plant-based preparations on diabetes.

**Systematic Review Registration:** https://www.crd.york.ac.uk/prospero/display_record.php?RecordID=220291, PROSPERO.

## Highlights

- Aloe vera, Psyllium fibre and Fenugreek seeds had the largest effects on HbA1c: −0.99, −0.97, and −0.85% respectively.
- Four other remedies reduced HbA1c by >0.5%, including *Nigella sativa* and *Astragalus membranaceus*.
- Tea (*Camellia sinensis*) and tea extracts were ineffective.
- No serious adverse effects were reported.

## Introduction

Type 2 Diabetes Mellitus (T2DM) is a major, growing health problem. It is estimated that 9.3% of the world’s population (463 million people) were living with diabetes in 2019 and this is projected to increase to 10.2% (578 million) by 2030 and 10.9% (700 million) by 2,045 (Saeedi et al., 2019). Over 90% of these have T2DM and over 1 million deaths per year are attributable to diabetes (Khan et al., 2020). The costs are huge: the USA alone spends $294 billion per year on management of diabetes in the population aged 20–79 (International Diabetes Federation, 2019).

Initial treatment of diabetes involves lifestyle modifications including changes to the diet and increasing physical activity, but dietary advice does not usually extend to herbs and phytomedicines. On average, compared to normal diets, low carbohydrate diets reduce HbA1c by only 0.09% (1 mmol/mol) (Korsmo-Haugen et al., 2019). Individualised dietary advice is recommended alongside a personalised management plan that aims to reduce and maintain HbA1c to below 6.5% (National Institute for Health and Care Excellence, 2020). Pharmacotherapy is initiated if patients fail to maintain HbA1c levels below this threshold.

Among adults with T2DM, 45% had not achieved adequate glycaemic control, in a national cross-sectional survey in the USA (Wong et al., 2013); poor adherence to medications is a major reason (Polonsky and Henry, 2016). Less than 50% of patients prescribed metformin were adherent and a third discontinued within 12 months, in a retrospective study in the UK Clinical Practice Research Datalink database (CPRD) (Tang et al., 2020). Side-effects of medication are the commonest reason for non-adherence (Grant et al., 2003). As many as 62% of patients taking metformin complain of diarrhoea (Florez et al., 2010).

Diabetes mellitus has been recognised for thousands of years and treated by traditional systems of medicine in Egypt, China, India, and Africa (Simmonds et al., 2006). Many patients with diabetes still use complementary therapies, ranging from 17% in the UK to 72% in the USA (Chang et al., 2007). Herbal medicines are among the most popular: they are used by 68% of diabetic patients in Saudi Arabia (Alqathama et al., 2020), 62% in Mexico (Chang et al., 2007), 62% in Ethiopia (Mekuria et al., 2018) and 58% in Sudan (Ali and Mahfouz, 2014). In India, 67% of diabetic patients use naturopathy or Ayurveda (Chang et al., 2007). However, the majority do not inform their doctors about their use of herbal medicine (Mekuria et al., 2018; Alqathama et al., 2020). In a qualitative study of members of the Pakistani community in Bradford (UK), two-thirds preferred using herbal medicine compared to conventional medicine and many believed that the vegetable “Karela” (*Momordica charantia*) could cure diabetes (Pieroni et al., 2008). Worldwide, about 1,200 plant species are reportedly used for the treatment of diabetes (Simmonds et al., 2006).

Although there has been a wealth of laboratory and clinical research on herbal medicines for diabetes, this has not been translated into user-friendly evidence-based information to guide patients or clinicians. Most patients base their choice of herbal medicines on advice from family and friends (Ali and Mahfouz, 2014; Mekuria et al., 2018). Although there have been several systematic reviews about herbal medicines for diabetes (Yeh et al., 2003; Wang et al., 2013; Gupta et al., 2017; Governa et al., 2018), none has yet provided a ranking of remedies for their effectiveness on glycaemic control in patients with T2DM. We aim to determine the relative effectiveness of common herbal remedies for treatment of type 2 diabetes through a systematic overview of meta-analyses of controlled clinical trials.

## Methods

The protocol for this study was registered on PROSPERO prior to starting data extraction: https://www.crd.york.ac.uk/prospero/display_record.php?RecordID=220291.

The protocol included the research question, search strategy, inclusion criteria and quality appraisal.

### Data Sources and Searches

We searched the following databases on October 7, 2020 for systematic reviews of randomised controlled clinical trials: EMBASE via OVID (from 1947), MEDLINE via OVID (from 1946), CINAHL (Cumulated Index to Nursing and Allied Health Literature from 1977) and the Cochrane Library including the Cochrane Central Register of Controlled Trials (CENTRAL). Each search strategy was adapted to take into account differences in controlled vocabulary and syntax rules. An example search strategy is given in Supplementary Material. We also contacted experts in the field to identify any relevant studies which had not been found by the search.

### Study Selection

Two reviewers independently screened titles and abstracts to select articles for full-text screening. Two reviewers then independently screened the full-text articles. We selected articles which met the following inclusion criteria:

- Study type: Systematic reviews of randomised controlled clinical trials with a reported systematic search strategy and with the intention to perform a meta-analysis.
- Participants: human subjects diagnosed with Type 2 diabetes, both diet-controlled and those on oral hypoglycaemic medications.
- Interventions: one specific herb or standardised herbal remedy
- Comparison: An inactive treatment (placebo) or standard care (oral hypoglycaemic medications, conventional diets)
-Outcomes: quantified change in HbA1c and/or fasting plasma glucose (FPG), reported as a numerical effect size.

We excluded reviews which only presented results in a narrative format and did not attempt to meta-analyse the outcomes. We did however include systematic reviews which found only a single relevant trial and presented its results in the correct format–where a meta-analysis had been intended but included only a single trial. Some reviews included trials both on T2DM and also on prediabetes. If results for T2DM were presented separately, and/or if trials in T2DM were the majority of included trials, we included these. We excluded reviews where the majority of included trials were not on patients with T2DM and where it was not possible to separate out the results for T2DM patients. We also excluded reviews where results for type 1 diabetes (T1DM) were not presented separately. We excluded reviews of multiple different herbal remedies and of pure compounds extracted from herbs, because none of these presented meta-analyses of individual medicinal plants. We did not apply any language restrictions.

### Data Extraction and Quality Assessment

Two reviewers independently extracted relevant data using a data extraction form created on Microsoft Excel, and any discrepancies were checked by a third reviewer. Where a review reported several patient groups and/or outcomes, we extracted the number of trials and participants which matched our inclusion criteria (type 2 diabetes) and which reported each relevant outcome (HbA1c and FPG). When results were presented separately for different types of control, we preferentially chose the comparison against placebo (rather than comparison against standard treatment), in order to gauge the effect size of the medicinal plant itself. Where HbA1c results were reported in mmol/mol, they were multiplied by the conversion factor 0.09148 to give the equivalent as a percentage (National Glycohemoglobin Standardization Program, 2010). Where FPG results were presented as mg/dL, they were divided by 18 to convert to mmol/L. For each review we extracted the number of trials which had reported on adverse effects, and the number of these which reported any specific adverse effects.

Two reviewers independently appraised the quality of the studies using the AMSTAR-2 tool (Shea et al., 2017) and discrepancies were resolved by discussion with a third reviewer.

### Data Synthesis and Analysis

Results from meta-analyses of HbA1c and FPG were ranked in order of effect size and presented on a Forest plot. A clinically significant reduction in HbA1c has been defined by clinicians as a reduction of at least ≥0.5% (Lenters-Westra et al., 2014); we defined a clinically significant reduction in FPG as a change of 0.5 mmol/l or more. We conducted a narrative synthesis of the other results. We calculated the Spearman’s rank correlation coefficient for the correlation between rank of effect on HbA1c and FPG. In this analysis we only included remedies for which both measures were reported. Where a remedy had differing results from several reviews, we took the rank of the best result for each of HbA1c and FPG.

## Results

### Included Studies

**FIGURE 1:**
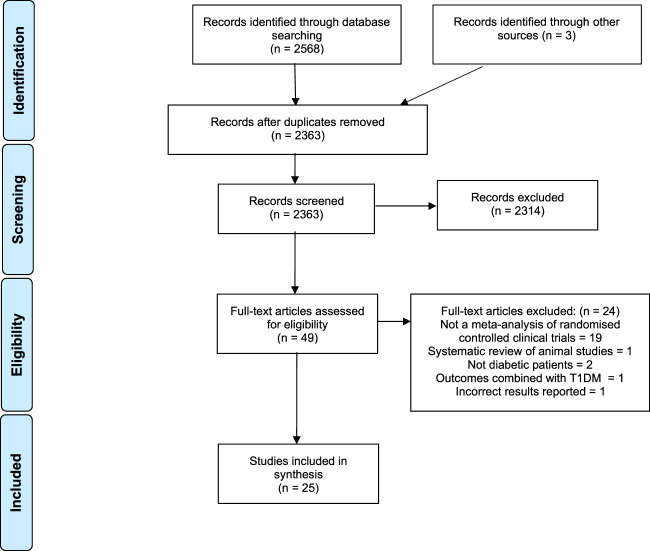
PRISMA flowchart.

Our initial search identified 2,363 articles after removing duplicates (Figure 1). Forty-nine full texts were screened and of these, 25 met all our inclusion criteria (Davis and Yokoyama, 2011; Kim et al., 2011; Leach and Kumar, 2012; Ooi et al., 2012; Allen et al., 2013; Ooi and Loke, 2013; Neelakantan et al., 2014; Gibb et al., 2015; Gui et al., 2016; Li et al., 2016; Shin et al., 2016; Suksomboon et al., 2016; Tian et al., 2016; Zhang et al., 2016; Daryabeygi-Khotbehsara et al., 2017; Poolsup et al., 2017; Schwingshackl et al., 2017; Gu et al., 2018; Deyno et al., 2019; Gao et al., 2019; Huang et al., 2019; Namazi et al., 2019; Peter et al., 2019; Yang et al., 2019; Ziaei et al., 2020). The commonest reason for exclusion was that the review did not attempt a quantitative meta-analysis of randomised controlled trials. One of the meta-analyses was excluded because it had incorrectly reported underlying data from included studies and its results were inaccurate (Gong et al., 2016).

There were reviews on 18 different medicinal plants (Table 1). Some herbal remedies had more than one review: cinnamon (Davis and Yokoyama, 2011; Leach and Kumar, 2012; Allen et al., 2013; Deyno et al., 2019; Namazi et al., 2019), ginseng (Kim et al., 2011; Gui et al., 2016), *Aloe vera* (Suksomboon et al., 2016; Zhang et al., 2016) and karela (*Momordica charantia*) (Ooi et al., 2012; Peter et al., 2019). Three reviews evaluated the effect of a standard traditional Chinese herbal formula which contained a mixture of several herbs. Gegen formulae contained *Pueraria lobata* root as their main constituent alongside other ingredients such as *Salvia miltiorrhiza* root, liquorice root and *Dioscorea opposita* rhizome (Yang et al., 2019). Jinqi Jiangtang contains *Astragalus membranaceus* root, *Coptis* spp rhizome and *lonicera japonica* (Gao et al., 2019). Tianmai Xiaoke contains *Trichosanthes* root, *Ophiopogon japonicus* root, *Schisandra chinensis* fruit and chromium picolinate (Gu et al., 2018). Some reviews studied the effect of specific plant products which are also used as foods: olive oil (Schwingshackl et al., 2017), sweet potato (Ooi and Loke, 2013), dragon fruit (Poolsup et al., 2017), and fenugreek powder incorporated into chapatis (Neelakantan et al., 2014).

**TABLE 1:** Characteristics of included studies.

Herbal remedy	References	Plant species	Plant part	Preparation	Daily dose (mg)	Control	Concomitant treatment (both groups)	Patient type	Follow-up duration for measuring outcomes (weeks)	Study types included	Number of trials	Total number of participants
Aloe vera	Suksomboon et al., (2016)	*Aloe vera* (L) Burm. F. (Xanthorrhoeaceae)	leaf	raw/juice/gel powder	600–30,000	Placebo/no treatment	OHA/insulin	T2DM	8–12	RCTs	5	235
Aloe vera	Zhang et al., (2016)	*Aloe vera* (L) Burm. F. (Xanthorrhoeaceae)	leaf	juice/powder	200–2,800	Placebo	None	T2DM + prediabetes	6–12	RCTs	5	415
Astragalus	Tian et al., (2016)	*Astragalus membranaceus* (Fisch) Bunge (Fabaceae)	root	aqueous decoction/injection	1,200–320,000	No treatment	OHA	T2DM	2–16	RCTs	13	1,054
Cinnamon	Allen et al., (2013)	*Cinnamomum cassia* (L.) J.Presl (Lauraceae)	Bark	aqueous extract/raw powder	120–6,000	Placebo	OHA	T2DM	4–18	RCTs	10	464
Cinnamon	Davis and Yokoyama, (2011)	*Cinnamomum cassia* (L.) J.Presl (Lauraceae)	Bark	powder/aqeuous extract	250–6,000	Placebo	OHA/none	T2DM + prediabetes	4–16	RCTs	8	369
Cinnamon	Deyno et al., (2019)	*Cinnamomum cassia* (L.) J.Presl and *Cinnamomum verum* J.Presl (Lauraceae)	NS	capsules	1,000–14,400	Placebo	OHA/none	T2DM + prediabetes	4–16	RCTs	16	1,098
Cinnamon	Leach and Kumar, (2012)	*Cinnamomum cassia* (L.) J.Presl and *Cinnamomum burmanni* (Nees & T.Nees) Blume (Lauraceae)	NS	tablet/capsule	500–6,000	Placebo	OHA/insulin	T1+T2DM	4–16	RCTs	10	304
Cinnamon	Namazi et al., (2019)	*Cinnamomum cassia* (L.) J.Presl (Lauraceae)	NS	Powder/extract	120–6,000	Placebo	None	T2DM	6–17	RCTs	18	1,100
Dragon Fruit	Poolsup et al., (2017)	*Hylocereus polyrhizus* (F.A.C.Weber) Britton & Rose; *Hylocereus costaricensis* (F.A.C.Weber) Britton & Rose (Cactaceae)	Fruit	Fresh fruit	100,000–600,000	No treatment	NS	T2DM	2–4	RCTs	2	58
Fenugreek	Neelakantan et al., (2014)	*Trigonella foenum-graecum* L (Fabaceae)	seed	powder/extract in capsules/chapati	1,000–100,000	Placebo	OHA	T2DM	1.5–12	9 RCTs and 1 controlled trial	10	278
Gegen formulae	Yang et al., (2019)	*Pueraria lobata* (Willd.) Ohwi (Fabaceae)	Root	mixture	NS	Placebo	OHA/insulin	T2DM	2–24	RCTs	13	1,440
Ginger	Huang et al., (2019)	*Zingiber officinale* Roscoe (Zingiberaceae)	NS	NS	1,600–4,000	NS	NS	T2DM	8–12	RCTs	8	454
Ginseng	Gui et al., (2016)	*Panax quinquefolius* L and *Panax ginseng* C.A.Mey (Araliaceae)	NS	Raw herb/hydrolysed extract in capsules	960–13,500	Placebo	Nil	Untreated early diabetes + prediabetes	4–20	RCTs	8	390
Ginseng	Kim et al., (2011)	*Panax ginseng* C.A.Mey (Araliaceae)	Root	Red Ginseng powder/fermented powder	780–3,000	Placebo	OHA	T2DM	12–24	RCTs	3	76
Jinqi Jiangtang	Gao et al., (2019)	*Astragalus membranaceus* (Fisch) Bunge (Fabaceae); *Coptis chinensis* Franch. (Ranunculaceae); *Lonicera japonica* Thunb. (Caprifoliaceae)	Root (*Astragalus* and *Coptis*), Flower (*Lonicera*)	Tablets	2,520–16,800	No treatment	OHA	T2DM	3–26	RCTs	17	1,365
Momordica charantia	Ooi et al., (2012)	*Momordica charantia* L (Cucurbitaceae)	fruit	dried powder in capsules	3,000	Placebo, OHA	Diet only	T2DM	12	RCTs	1	40
Momordica charantia	Peter et al., (2019)	*Momordica charantia* L (Cucurbitaceae)	Fruit	dried pulp/juice	1,200–6,000	Placebo, OHA	OHA/none	T2DM	4–16	RCTs	6	243
Mulberry	Shin et al., (2016)	*Morus alba* L. (Moraceae)	leaf	Extract in capsules	1,000	Placebo, OHA	None	T2DM	12	RCTs	1	23
Nettle	Ziaei et al., (2020)	*Urtica dioica* L (Urticaceae)	NS	NS	1,500–10,000	placebo	None	T2DM	8–12	RCTs	8	266
Nigella sativa	Daryabeygi-Khotbehsara et al., (2017)	*Nigella sativa* L (Ranunculaceae)	seed	powder/oil	500–2000mg; 1–5 ml	Placebo	OHA/none	T2DM	8–52	4 RCTs and 3 non-randomised controlled trials	7	505
Olive Oil	Schwingshackl et al., (2017)	*Olea europaea* L. (Oleaceae)	fruit	oil	10,000–75,000	Low-fat diet/fish oils/PUFA	NS	T2DM	2–208	RCTs	25	1724
Psyllium Fiber	Gibb et al., (2015)	*Plantago psyllium* L.; *Plantago ovata* Forssk. (Plantaginaceae)	Seed	husk	6,800–15,000	Placebo/no treatment	None	T2DM	6–12	RCTs	4	245
Sweet Potato	Ooi and Loke, (2013)	*Ipomoea batatas* (L.) Lam. (Convolvulaceae)	Rhizome	Dry powder in tablets	4,000	Placebo	None	T2DM	6–20	RCTs	2	122
Tea	Li et al., (2016)	*Camellia sinensis* (L.) Kuntze (Theaceae)	Leaf	green/black/oolong tea/capsules	150–1,500	Placebo/water	NS	T2DM	4–16	RCTs	12	658
Tianmai Xiaoke	Gu et al., (2018)	*Trichosanthes kirilowii* Maxim. (Cucurbitaceae); *Ophiopogon japonicus* (Thunb.) Ker Gawl. (Asparagaceae); *Schisandra chinensis* (Turcz.) Baill. (Schisandraceae)	Root (*Trichosanthes*, Ophiopogon); Fruit (Schisandra)	Tablets, also containing chromium picolinate (1.6 mg per tablet)	480	No treatment	OHA/insulin	T2DM	8–16	RCTs	7	717

Abbreviations: OHA, oral hypoglycaemic agent; T1DM, Type 1 Diabetes Mellitus; T2DM, type 2 diabetes mellitus; NS, Not Specified; RCTs, Randomised Controlled Trials.

All the reviews included mainly clinical trials in patients with T2DM (see Table 1) but four also included a few trials in patients with pre-diabetes. One included a single trial in patients with T1DM, but its results were presented separately and excluded from this review. Five reviews included only trials of patients with diet-controlled diabetes, not taking any conventional antidiabetic medications. Fourteen reviews included trials in which both intervention and control groups received concomitant conventional treatment with oral hypoglycaemic agents (OHA). Five reviews did not specify whether concomitant treatment was given. In 19 reviews, the control groups received a placebo, in four reviews they received only the conventional care (diet and/or medications) and in one, some control groups received a fish oil supplement. In three reviews, some studies gave a conventional OHA to the control group only, not to the treatment group (Ooi et al., 2012; Shin et al., 2016; Peter et al., 2019) but for this review we only extracted the outcomes from the studies using a placebo control.

Duration of follow-up was most often 4–12 weeks, but there was a wide range with a few included studies following up for as little as 1 week or for as long as 4 years. All the reviews included randomised controlled trials but two also included a few non-randomised controlled trials. The reviews included a median of eight trials and 390 participants but the smallest included only a single trial and the largest review included 25 studies (1724 participants).

### Quality Assessment

The AMSTAR-2 scores for each study are shown in Supplementary Material. Only three reviews scored “yes” on all the criteria–all of them Cochrane reviews (Leach and Kumar, 2012; Ooi et al., 2012; Ooi and Loke, 2013). Several quality issues were identified with the other reviews. Most did not report that there was a pre-established published protocol. Most did not have a fully comprehensive search strategy including the grey literature. Most did not list all excluded studies and most did not report on the sources of funding for the studies included in the review. Seven did not adequately investigate publication bias. Six did not report conflicts of interest, including the review on Psyllium which was led by a company marketing a Psyllium product (Gibb et al., 2015)—this review is at high risk of bias.

### Effect Size on HbA1c

**FIGURE 2:**
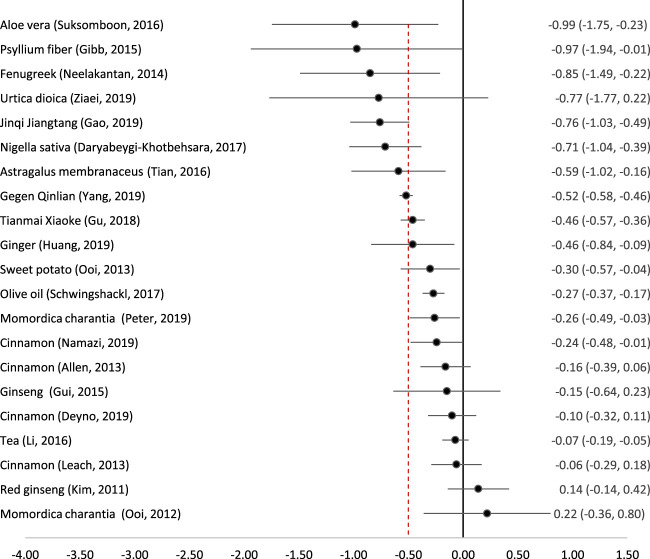
Effect of medicinal plants on HbA1c (%). The red dotted line indicates the threshold for a clinically significant effect (reduction by 0.5%). Point indicates the effect size, and the line (and figures to the right) indicate the 95% confidence interval.

Twenty-one studies on 16 remedies attempted to conduct a meta-analysis quantifying the reduction in HbA1c (Figure 2). The most effective remedy appeared to be Aloe vera (freshly extracted juice) (Suksomboon et al., 2016). Psyllium fibre (Gibb et al., 2015) and Fenugreek seeds (Neelakantan et al., 2014) also led to similar reductions in HbA1c although with wider confidence intervals. *Nigella sativa* seeds (Daryabeygi-Khotbehsara et al., 2017), *Astragalus membranaceus* root (Tian et al., 2016), and two complex traditional Chinese formulae (Gegen Qinlian (Yang et al., 2019) and Jinqi Jiantang (Gao et al., 2019)) also led to clinically and statistically significant reductions in HbA1c. Nettle (*Urtica dioica*) appeared to lead to a clinically significant reduction but this was not statistically significant because of very wide confidence intervals (Ziaei et al., 2020).

Several remedies produced a statistically significant reduction in HbA1c but the standard mean difference fell below the pre-determined threshold of 0.5%. These were the patent traditional Chinese formula Tianmai Xiaoke (Gu et al., 2018), ginger (Huang et al., 2019), sweet potato tablets (Ooi and Loke, 2013), olive oil (Schwingshackl et al., 2017), karela (*Momordica charantia*) (Peter et al., 2019) and cinnamon (Namazi et al., 2019). *Momordica charantia* was studied by two reviews which came to differing conclusions; an early Cochrane review found only a single small RCT with 40 participants, which concluded that Karela dried powder in capsules appeared to be ineffective (Ooi et al., 2012). However, a more recent and comprehensive review including five RCTs (243 participants) found that there was a statistically significant reduction in HbA1c by 0.26% (Peter et al., 2019). Similarly, the four reviews on cinnamon which reported HbA1c came to slightly different conclusions; only one found a statistically significant reduction and none of them reported a clinically significant reduction.

Two meta-analyses of ginseng (Kim et al., 2011; Gui et al., 2016) and one of tea (*Camellia sinensis*) and tea extracts (Li et al., 2016) all showed that these remedies had no clinically or statistically significant effect on HbA1c.

### Effect Size on Fasting Plasma Glucose (FPG)

**FIGURE 3:**
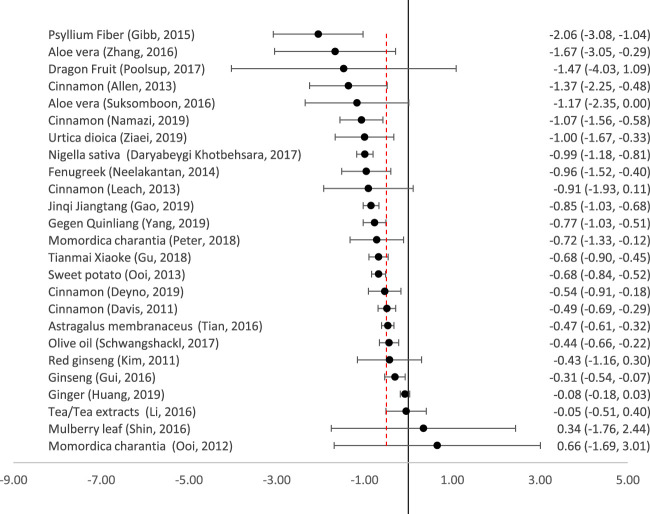
Effect of medicinal plants on Fasting Plasma Glucose (mmol/l). The red dotted line indicates the threshold for a clinically significant effect (reduction by 0.5mmol/l). The point indicates the effect size, and the line (and figures to the right) indicate the 95% confidence interval.

Twenty-five reviews meta-analysed the reduction in FPG (Figure 3). All the remedies which produced clinically significant reductions in HbA1c also produced clinically and statistically significant reductions in FPG, with the exception of *Astragalus membranaceus*, which reduced FPG slightly less than the predetermined clinically significant threshold of 0.5 mmol/l. Nettle (*Urtica dioica*), *Momordica charantia* and sweet potato also all produced clinically significant reductions in FPG.

There were varying results in the five meta-analyses of cinnamon, but the largest and most recent (which only included patients with T2DM) showed a clinically significant reduction in FPG of −1.07 mmol/l (95% CI-1.56 to −0.58) (Namazi et al., 2019). Other reviews also included patients with T1DM (Leach and Kumar, 2012) or pre-diabetes (Davis and Yokoyama, 2011; Deyno et al., 2019).

For several remedies, there was a wide degree of uncertainty regarding their effectiveness in reducing FPG. Dragon fruit appeared to have a large effect but this was not statistically significant as there were very wide confidence intervals (Poolsup et al., 2017). There was also a large degree of uncertainty about the effect of Mulberry leaf–there was a wide confidence interval, and a second trial (not included in the meta-analysis) reported that it was more effective than glibenclamide (Shin et al., 2016). Of the two reviews on Ginseng, that by Kim et al. (2011) was the only one to focus purely on T2DM; it showed a non-significant reduction in FPG. Another meta-analysis did report a significant reduction in FPG but also included pre-diabetic patients (Gui et al., 2016).

It can be stated with some certainty that ginger and tea (*Camellia sinensis*) extracts were ineffective for reducing FPG. Neither had a significant effect, and confidence intervals were tight.

### Correlation Between Effect on HbA1c and FPG

Spearman’s rank correlation coefficient was 0.70, indicating that there was a moderate correlation between effect on HbA1c and FPG.

### Adverse Effects

None of the included reviews reported any serious adverse events. In most cases there was no significant difference in the incidence of adverse events between the treatment and control groups (Table 2). Mild gastrointestinal symptoms such as diarrhoea, vomiting and abdominal discomfort were reported in a few cases for certain herbal remedies, in particular *Momordica charantia* (three participants) and Fenugreek seeds (three participants). There was no specific mention of drug interactions although 14 of the reviews included trials in which the herbal medicine was given in addition to conventional oral hypoglycaemic agents. Only three of these reviews mentioned cases of hypoglycaemia, including only one reported case of a hypoglycaemic seizure in a clinical trial of cinnamon given to adolescent T1DM patients on insulin (Leach and Kumar, 2012).

**TABLE 2:** Reported adverse effects.

Herbal remedy	References	Number of trials	Adverse effects (N of trials reporting)
*Aloe vera*	Suksomboon et al., (2016)	5	None (4); diarrhoea/vomiting (1)
*Aloe vera*	Zhang et al., (2016)	5	Only one trial reported one adverse event (not specified)
*Astragalus*	Tian et al., (2016)	13	None (3)
Cinnamon	Allen et al., (2013)	10	No significant adverse effects (10)
Cinnamon	Davis and Yokoyama, (2011)	8	Not reported
Cinnamon	Deyno et al., (2019)	16	“Well tolerated"
Cinnamon	Leach and Kumar, (2012)	10	No difference in incidence between treatment and control (3). One case of hives, and one of hypoglycaemic seizure (in a trial in T1DM patients on insulin).
Cinnamon	Namazi et al., (2019)	18	One case of “skin allergy” after taking the remedy for 90 days.
Dragon Fruit	Poolsup et al., (2017)	2	None (2)
Fenugreek	Neelakantan et al., (2014)	10	Mild gastrointestinal symptoms (3)
Gegen Qinlian	Yang et al., (2019)	13	None reported (15); fewer than in control group (12); mild g-i effects (2)
Ginger	Huang et al., (2019)	8	Not reported
Ginseng	Gui et al., (2016)	8	No serious adverse events (8)
Ginseng	Kim et al., (2011)	3	No difference in incidence compared to control; reports of tachycardia, headache, blurry vision, insomnia, irritability and hypoglycemia (1)
Ginseng	Shishtar et al., (2014)	16	No difference in incidence compared to control (4)
Jinqi Jiangtang	Gao et al., (2019)	17	No difference in incidence compared to control (8)
*Momordica charantia*	Ooi et al., (2012)	1	No serious adverse events (3); gastrointestinal symptoms (2)
*Momordica charantia*	Peter et al., (2019)	6	Gastrointestinal symptoms (5), headache/dizziness (2), rash (1), sore throat (1), hypotension (1)
Mulberry	Shin et al., (2016)	1	Not reported
Nettle	Ziaei et al., (2020)	8	No significant adverse events (7), itching (1)
*Nigella sativa*	Daryabeygi-Khotbehsara et al., (2017)	7	None (6); mild g-i side effects (1)
Olive Oil	Schwingshackl et al., (2017)	25	Not reported
Psyllium Fiber	Gibb et al., (2015)	4	Not reported
Sweet Potato	Ooi and Loke, (2013)	2	No difference in incidence compared to control (2)
Tea	Li et al., (2016)	12	Not reported
Tianmai Xiaoke	Gu et al., (2018)	7	Gastrointestinal symptoms, nervous system symptoms, and hypoglycemia (7)

## Discussion

### Summary of Main Findings

There have been many RCTs on different phytomedicines and herbal medicines for T2DM, and 25 published meta-analyses on 18 different medicinal plants. Of these, seven have a clinically and statistically significant effect on HbA1c and 12 on FPG (Figures 2, 3). The most effective on both measures appear to be *Aloe vera*, Psyllium fibre, Fenugreek seeds, *Nigella sativa* seeds, and the complex traditional Chinese formula Jinqi Jiangtang. Tea and tea extracts were ineffective. The 12 other remedies showed some degree of effectiveness on either HbA1c or FPG, but in some cases with a wide degree of uncertainty. All of the medicinal plants evaluated in this review appeared to be safe, with no serious adverse effects reported. However, some were associated with minor side-effects, in particular gastrointestinal disturbances.

### Strengths and Limitations

This the first study to provide a systematic, evidence-based overview of meta-analyses of the effectiveness of medicinal plants for glycaemic control in type 2 diabetes. Our systematic approach with broad search terms ensured that we probably found most relevant articles. One limitation is that we did not have the time to search the grey literature or databases in foreign languages such as Chinese. Another limitation is that we were not able to include medicinal plants for which there had been no systematic review with a meta-analysis. For example there was a systematic review of the Ayurvedic remedy *Gymnema sylvestre* (Leach, 2007) but this found no clinical trials which met its inclusion criteria. It is also likely that there are other potentially effective medicinal plants which have been evaluated in RCTs but not reviewed in a meta-analysis, and others which have not been evaluated in an RCT although lower-level evidence suggests they could be effective (Sissoko et al., 2020).

Our results are also limited by the quality of the trials included in the meta-analyses. Although most only included RCTs, in some cases the preparation or dosage of the phytomedicine may have been suboptimal; in some reviews both herbal remedies and standardised phytomedicines were included. The clinical condition of the patients may have been different between trials where patients were taking concomitant oral antidiabetics and those who were purely diet controlled. In some trials, the duration of follow-up was insufficient to measure the effect on HbA1c, which should be measured at least 3 months after the start of treatment to reveal its full effect. Follow-up duration was generally short: only three reviews included studies with follow-up of 1 year or more, so there is little information on long-term adherence to herbal remedies.

### Comparison With the Existing Literature

The effect of the most promising medicinal plants was similar to that of standard oral hypoglycaemic agents. In a meta-analysis, metformin monotherapy lowered HbA1c by 1.12% (95% CI 0.92–1.32) versus placebo. Metformin added to oral therapy lowered HbA1c by 0.95% (0.77–1.13) versus placebo added to oral therapy (Hirst et al., 2012). In another meta-analysis, metformin reduced FPG by −2.0 mmol/l (95% CI: −2.4, −1.7) (Johansen, 1999). Other conventional hypoglycaemic medications have a smaller effect, for example sitagliptin lowers HbA1c by −0.94% and FPG by 1.2 mmol/l (Aschner et al., 2006).

Several mechanisms of action explain the effect of medicinal plants. Firstly, many plant products contain gel-forming fibres which delay gastric emptying and interfere with glucose absorption from the intestines–for example *Aloe vera* (Suksomboon et al., 2016), Fenugreek (Madar and Shomer, 1990) and Psyllium (Gibb et al., 2015). Secondly, some medicinal plants contain substances which inhibit enzymes involved in digestion of carbohydrates (eg α-amylase, α-glucosidase), such as nettle (Ziaei et al., 2020) and the Chinese formula Jinqui Jiangtan (Gao et al., 2019). Third, others stimulate release of insulin; these include Fenugreek seeds (Neelakantan et al., 2014) and *Nigella sativa* seeds (Daryabeygi-Khotbehsara et al., 2017). Fourth, some medicinal plants inhibit gluconeogenesis, including *Nigella sativa* (Daryabeygi-Khotbehsara et al., 2017). Fifth, some, such as nettle (Ziaei et al., 2020), mimic the effect of insulin by increasing peripheral uptake of glucose, while others such as *Nigella sativa* induce insulin sensitivity (Daryabeygi-Khotbehsara et al., 2017).

### Implications for Policy and Practice

Dietary and lifestyle advice for patients with diabetes rarely includes information on natural remedies, herbs and spices that can help with glycaemic control. The results presented here can guide patients who wish to try herbal supplements and foods as part of their self-care and diet, and clinicians who wish to advise them. Several of the remedies tested are effective and safe. Many of these herbs and spices with clinically assessed hypoglycemic properties are common food products, and as such generally considered very safe. Some can easily be incorporated into the diet–for example in some studies fenugreek seed powder was mixed with flour for baking chapatis, to reach a total daily dose of 100 g (Neelakantan et al., 2014); but the most effective preparation appeared to be a standardised extract of Fenugreek seed total saponins given in six capsules three times daily after meals (Lu et al., 2008). Other herbs can easily be purchased without a prescription (for example *Aloe vera*, Psyllium fibre, and *Nigella* seeds). However, it would be necessary to ensure that an adequate dosage is taken of the most effective preparations. The most effective preparation of *Aloe vera* appeared to be freshly extracted juice, followed by powdered gel in capsules (Suksomboon et al., 2016). In the case of Psyllium, the most effective preparation appeared to be the seed husk of *Plantago ovata* Forssk (Ziai et al., 2005). For *Nigella sativa*, the seed powder (at a dose of 2 g daily) was more effective than the oil (Daryabeygi-Khotbehsara et al., 2017). It is equally important to inform patients and clinicians about remedies which appear to be ineffective–such as tea extracts–and those for which there is insufficient evidence of effectiveness–for example cinnamon and ginseng.

### Priorities for Future Research

Firstly, some of the meta-analyses were performed more than 5 years ago and need to be updated to include the most recent trials. Some reviews were not performed to the highest standards and could be improved. In particular we recommend that the meta-analysis on Fenugreek should be updated because this appears to be one of the most effective remedies but the systematic review was done in 2014 (Neelakantan et al., 2014). A later systematic review suggested an even greater effect but incorrectly reported some of the underlying data (Gong et al., 2016). It would also be useful to perform a network meta-analysis to estimate the relative effects between the different herbal interventions.

Secondly, it would be interesting to evaluate the impact on glycaemic control of including information on effective medicinal plants and herbal remedies within dietary and lifestyle advice for patients with type 2 diabetes. These may have an additional benefit, and for some patients may be more acceptable, so may be a useful addition to the “menu” of options. This information would need to include clear instructions on the most effective preparations and dosages, and to warn patients about potential side-effects.

Thirdly, this review found a large number of potentially effective medicinal plants for which there is insufficient evidence of effectiveness. For example, Nettle (*Urtica dioica*) appears to have a significant effect on HbA1c and FPG (Ziaei et al., 2020) but the confidence intervals are very wide. Larger trials are needed to provide a more precise estimate of efficacy. Although it appears effective, the results on Psyllium were at high risk of bias because the review was undertaken by a company selling it–a higher quality review, with low risk of bias, would be helpful. In some studies, cinnamon appears to significantly reduce FPG, but not HbA1c. However, there is a wide variety of cinnamon species, preparations and doses–it is likely that some are more effective than others. Further research is needed to identify the most effective preparations and dosages, and to conduct high-quality clinical trials of these.

Fourth, for the majority of the 1,200 remedies which have been traditionally used in the treatment of diabetes (Simmonds et al., 2006), no meta-analyses and/or no RCTs have been conducted. Some of these have preliminary evidence of effectiveness, for example on post-prandial glucose; these include the Ayurvedic remedy *Gymnema sylvestre* (Leach, 2007) and the West African tree *Moringa oleifera* (Sissoko et al., 2020). It is important to conduct high-quality clinical trials of these (at low risk of bias, using a standardised, replicable dosage and preparation, and measuring HbA1c after at least 12 weeks).

## Conclusion

Several medicinal plants have the potential to lower HbA1c and could be effective as an adjunct to other lifestyle measures and current treatment, in particular Aloe vera, Psyllium fibre, Fenugreek seeds, Nigella sativa seeds and the Chinese formula Jinqi Jiangtang. It is also clear that tea and tea extracts are ineffective. Rigorous trials with at least 3 months follow-up are needed to ascertain the safety and effectiveness of promising plant-based preparations on diabetes. Practical information on safe plant-based preparations with hypo-glycaemic effects should be made widely available to clinicians and patients with diabetes.

## Data Availability

The original contributions presented in the study are included in the article/Supplementary Material, further inquiries can be directed to the corresponding author.
