# Supplementary material for: Effectiveness of Medicinal Plants for Glycaemic Control in Type 2 Diabetes: An Overview of Meta-Analyses of Clinical Trials
Source: Front Pharmacol. 2021 Nov 26;12:777561. doi: 10.3389/fphar.2021.777561 (PMC8662558; doi:10.3389/fphar.2021.777561)
Supplement: Supplementary file 1 [file DataSheet1.docx]

# Appendix 1: Search strategies

## Cochrane Central Register of Controlled Trials (CENTRAL) via Cochrane Library

1. Diabetes Mellitus, Type 2 explode all trees

2. Insulin Resistance explode all trees

3. (glucose and intoleran*) OR (insulin* and resistan*)

4. MODY OR NIDDM OR T2DM OR TDM2

5. #1 OR #2 OR #3 OR #4

6. Phytotherapy explode all trees

7. Herb* OR Spic*

8. Medicinal teas

9. Plants, medicinal

10. Exp ayurvedic medicine

11. Exp diet therapy

12. Exp Medicine, traditional

13. Traditional Chinese Medicines

14. (herbal or Chinese or ayurved*) adj2 (therap* Or medicine Or treatment Or intervention).ti,ab.

15. #6 OR #7 OR #8 OR #9 OR #10 OR #11 OR #12 OR 13 OR 14

16. #5 and #15

17. Exp systematic review

18. #16 AND #17

## CINAHL (Cumulated Index to Nursing and Allied Health Literature) from 1977

1. Herb* or spic*

2. Phytotherapy

3. Ayurvedic Medicine

4. Traditional chinese medicine

5. Traditional medicine

6. Diet therapy

7. Medicinal tea

8. OR #1 - #7

9. Diabetes Mellitus, Type 2

10. Diabet* AND (Type 2 OR type two OR type II)

11. Insulin Resistance

12. (MODY) or (NIDDM) OR (T2DM)

13. Non insulin* depend* OR noninsulin* depend*

14. Diabet* N3 (type 2 or type II)

15. (onset N3 (late or adult* or matur* or slow or stabl*)) and AB diabet*

16. (‘insulin*defic*” N3 relativ*)

17. (Insulin* resistan*)

18. OR #9 - #17

19. #8 AND #18

20. Systematic review

21. Systematic literature review

22. Meta-analysis

23. Comparative study

24. OR #20 - #23

25. #19 AND #24

## EMBASE via Ovid from 1947

1. Herb* or spic*.mp.

2. Phytotherapy.mp. or exp phytotherap*/

3. Ayurvedic medicine.mp. or Ayurveda/

4. Traditional Chinese Medicine.mp. or Chinese medicine

5. Traditional medicine

6. Diet therapy

7. Medicinal teas

8. Plants, medicinal/

9. (herbal or Chinese or ayurved*) adj2 (therap* or medicine? Or treatment? Or intervention?)).ti,ab.

10. OR #1 - #9

11. Non insulin dependent diabetes mellitus.mp. or non insulin dependent diabetes mellitus/

12. Exp insulin resistance/

13. (MODY or NIDDM or T2D or T2DM).mp.

14. ((type 2 or type II or type two) adj3 diabet*).mp.

15. (obes* adj3 diabet*).mp.

16. (non insulin* depend* or noninsulin* depend*).mp.

17. ((adult* or matur* or late or slow or stabl*) adj3 diabet*).mp.

18. Insulin* resistanc*.mp.

19. OR #11 - #18

20. #10 AND #19

21. Systematic review.mp.

22. Systematic literature review.mp.

23. Exp systematic review or systematic review.ti,ab.

24. Review.mp.

25. Comparative study.mp.

26. Meta-analysis.mp. or meta analyisis.ti,ab.

27. Outcome assessment.mp.

28. Systematic literature.mp.

29. Evidence synthesis.mp.

30. OR #21 - #29

31. NOT ((exp animal/or nonhuman/) NOT exp human/)

32. #20 AND #30 AND #31

## MEDLINE via Ovid from 1946:

1. Herb* or spic* .mp.

2. Phytotherapy.mp. or exp phytotherapy/

3. Ayurvedic medicine.mp. or exp medicine, ayurvedic/

4. Traditional Chinese Medicine

5. Traditional medicine

6. Diet therapy

7. Medicinal teas

8. Plants, medicinal/

9. (herbal or Chinese or ayurved*) adj2 (therap* or medicine? Or treatment? Or intervention?)).ti,ab.

10. OR #1 - #9

11. Type 2 diabetes.mp. or exp Diabetes Mellitus, Type 2/

12. (Type 2 diabetes or diabetes mellitus or type II diabetes mellitus).mp.

13. (type 2 adj diabetes).mp.

14. Diabetes mellitus.mp. or exp Diabetes Mellitus/

15. Exp insulin resistance/

16. Exp glucose metabolism disorders.

17. (NIDDM or T2DM).mp.

18. Insulin* defic*.mp.

19. Non insulin$ depend$ or noninsulin$ depend$.mp.

20. Impaired glucose$ toleranc$ or glucose$ intoleranc$ or insulin resistan$.mp.

21. ((late or adult$ or matur$ or slow or stabl$) adj3 onset) and diabet$).mp.

22. OR #11 - #21

23. #10 AND #22

24. Systematic review.ti,ab.

25. Exp ‘Systematic review’

26. Systematic literature review.ti,ab.

27. Comparative review.mp.

28. Review.mp.

29. Evidence synthesis.ti,ab.

30. Meta analysis.mp.

31. Meta analysis.ti,ab.

32. Exp meta analysis

33. Exp outcome assessment, health care/

34. OR #24 - #33

35. NOT (animals/ NOT (Animals/AND Humans/))

36. #23 AND #34 AND #35

# Appendix 2: Quality assessment of included studies (AMSTAR 2)

(Green = Yes; Yellow = Partial yes; Red = No)

| AMSTAR-2 item* | 1 | 2 | 3 | 4 | 5 | 6 | 7 | 8 | 9 | 10 | 11 | 12 | 13 | 14 | 15 | 16 |
| --- | --- | --- | --- | --- | --- | --- | --- | --- | --- | --- | --- | --- | --- | --- | --- | --- |
| Allen, 2013 | Y | N | Y | Partial Y | Y | Y | N | Partial Y | Y | N | Y | Y | Y | Y | Y | Y |
| Daryabeygi-Khotbehsara, 2017 | Y | N | N | Partial Y | Y | Y | N | Y | Y | N | Y | Y | Y | Y | Y | Y |
| Davis, 2011 | Y | N | Y | Partial Y | Y | N | Y | Y | N | N | N | N | N | N | Y | Y |
| Deyno , 2019 | Y | Y | Y | Partial Y | Y | N | Y | Partial Y | Y | N | Y | N | N | N | N | Y |
| Gao, 2019 | Y | Y | Y | Partial Y | Y | Y | N | Partial Y | Y | N | Y | Y | Y | Y | Y | N |
| Gibb, 2015 | Y | N | Y | Partial Y | Y | Y | N | Y | N | N | Y | N | N | N | N | N |
| Gu, 2018 | Y | Y | Y | Y | Y | Y | N | Y | Y | N | Y | Y | Y | Y | Y | Y |
| Gui, 2016 | Y | N | Y | Partial Y | Y | Y | N | Partial Y | Y | N | Y | Y | Y | Y | Y | Y |
| Huang, 2019 | Y | N | Y | Partial Y | N | Y | N | Partial Y | Y | N | Y | Y | N | Y | N | Y |
| Kim, 2011 | Y | N | Y | Partial Y | Y | Y | Y | Y | Y | N | Y | Y | Y | Y | Y | N |
| Leach, 2012 | Y | Y | Y | Y | Y | Y | Y | Y | Y | Y | Y | Y | Y | Y | Y | Y |
| Li, 2016 | Y | N | Y | Partial Y | Y | Y | N | Y | Y | N | Y | Y | Y | N | N | N |
| Namazi, 2019 | Y | N | Y | Partial Y | Y | Y | N | Y | Partial Y | N | Y | Y | N | N | Y | Y |
| Neelakantan, 2014 | N | N | N | Partial Y | Y | Y | N | Y | N | N | Y | N | N | Y | N | Y |
| Ooi, 2013 | Y | Y | Y | Y | Y | Y | Y | Y | Y | Y | Y | Y | Y | Y | Y | Y |
| Ooi, 2012 | Y | Y | Y | Y | Y | Y | Y | Y | Y | Y | Y | Y | Y | Y | Y | Y |
| Peter, 2019 | Y | Y | Y | Y | Y | N | Y | Y | Y | N | Y | Y | Y | Y | Y | Y |
| Poolsup, 2017 | Y | N | Y | Y | Y | Y | Y | Y | Y | N | Y | Y | Y | Y | N | Y |
| Schwingshackl, 2017 | Y | Y | Y | Partial Y | Y | N | N | Y | Y | N | Y | Y | N | N | Y | Y |
| Shin , 2016 | Y | N | Y | Partial Y | Y | Y | Y | Y | Y | Y | No meta-analysis | No meta-analysis | N | N | No meta-analysis | Y |
| Suksomboon, 2016 | Y | N | Y | Partial Y | Y | Y | Y | Y | N | N | Y | Y | Y | Y | N | Y |
| Tian, 2016 | Y | N | Y | Partial Y | Y | Y | N | Y | Y | N | Y | Y | Y | Y | Y | Y |
| Yang, 2019 | Y | N | Y | Partial Y | Y | Y | N | Partial Y | Y | N | Y | N | Y | Y | Y | N |
| Zhang, 2016 | N | N | Y | Partial Y | Y | Y | N | Partial Y | Partial Y | N | Y | N | N | Y | N | Y |
| Ziaei, 2019 | Y | N | Y | Partial Y | Y | Y | N | Y | Y | N | Y | Y | Y | Y | Y | Y |

*Explanation of the AMSTAR-2 criteria:

1: Did the research questions and inclusion criteria for the review include the components of PICO?

2: Did the report of the review contain an explicit statement/(protocol)?

3: Did the review explain selection of the study designs for inclusion in the review?

4: Did the review authors use a comprehensive literature search strategy?

5: Did the review authors perform study selection in duplicate?

6: Did the review authors perform data extraction in duplicate?

7: Did the review authors provide a list of excluded studies and justify the exclusions?

8: Did the review authors describe the included studies in adequate detail?

9: Did the review use a satisfactory technique for assessing the risk of bias (RoB) in individual studies that were included in the review?

10: Did the review authors report on the sources of funding for the studies included in the review?

11: If meta-analysis was performed, did the review authors use appropriate methods for statistical combination of results?

12: If meta-analysis was performed, did the review authors assess the potential impact of RoB in individual studies on the results of the meta-analysis or other evidence synthesis?

13: Did the review authors account for RoB in primary studies when interpreting/discussing the results of the review?

14: Did the review authors provide a satisfactory explanation for, and discussion of, any heterogeneity observed in the results of the review?

15: If they performed quantitative synthesis did the review authors carry out an adequate investigation of publication bias (small study bias) and discuss its likely impact on the results of the review?

16: Did the review authors report any potential sources of conflict of interest, including any funding they received for conducting the review?
